# Modelling the cost of engage & treat and test & treat strategies towards the elimination of lymphatic filariasis in Ghana

**DOI:** 10.1371/journal.pntd.0012213

**Published:** 2024-05-24

**Authors:** Nathaniel N. K. Adams, Collins S. Ahorlu, Dziedzom K. de Souza, Moses Aikins

**Affiliations:** 1 School of Public Health, College of Health Sciences, University of Ghana, Legon–Accra, Ghana; 2 Department of Epidemiology, Noguchi Memorial Institute for Medical Research, College of Health Sciences, University of Ghana, Legon–Accra, Ghana; 3 Department of Parasitology, Noguchi Memorial Institute for Medical Research, College of Health Sciences, University of Ghana, Legon–Accra, Ghana; Institute of Medical Microbiology, Immunology and Parasitology, GERMANY

## Abstract

**Background:**

Despite several years of LF-MDA implementation, Ghana still has some districts with mf prevalence >1%, partly due to poor treatment coverage levels resulting from non-participation in MDA. To address the challenges, we implemented Engage & Treat (E&T) and Test & Treat (T&T) strategies for individuals who miss or refuse MDA respectively, in a hotspot district, enabling us to reach many of those who seldom, or never, take part in MDA. This financial cost study was undertaken to analyse data on the LF-MDA, E&T and T&T implementation in 2021 and the financial cost to inform the rollout of the E&T and T&T as mop-up strategies in future LF-MDAs.

**Methods:**

This costing study analysed cost data from the 2021 LF-MDA implementation activities carried out by the Neglected Tropical Diseases (NTD) programme of the Ghana Health Service and the SENTINEL study, carried out in Ahanta West district for the two interventions (i.e., E&T and T&T). The 2021 Ghana Population and Housing Census data was used to estimate the LF-MDA-eligible population. The financial cost per person treated was estimated and these costs were applied to the projected population to obtain the financial cost for subsequent years.

**Results:**

Implementing MDA mop-up strategies either through the E&T or T&T to improve coverage comes at an additional cost to the elimination goals. For example, in 2024 the projected cost per person treated by the routine LF-MDA is estimated at US$0.83. The cost using the integrated LF-MDA and the E&T, T&T led by the NTD programme or T&T integrated into the health system was estimated at US$1.62, US$2.88, and US$2.33, respectively, for the same year. Despite the increased cost, the proposed combined LF-MDA and mop-up strategies will have a higher estimated population treated for 2024 (i.e., 1,392,211) compared to the routine LF-MDA approach (i.e., 988,470) for the same year.

**Conclusion:**

Combining LF-MDA with E&T/T&T mop-up strategies, despite their high costs, may provide NTD Programmes with the options of improving treatment coverage and reaching the LF elimination target sooner, given that the routine LF-MDA alone approach has been implemented for many years with some districts yet to reach the elimination targets.

## Author summary

In Ghana, despite many years of efforts to eliminate lymphatic filariasis (LF) through mass drug administration (MDA), some districts still have a prevalence of infection above the threshold required to eliminate the disease as a public health problem. This is partly because not everyone in these districts is participating in the MDA programme. To tackle this challenge, new strategies called Engage & Treat (E&T) and Test & Treat (T&T) were implemented in a hotspot district. These strategies focus on reaching individuals who either miss or refuse MDA, ensuring that even those who rarely or never participate are covered. A financial cost study was undertaken to analyze the data from LF-MDA, E&T, and T&T implementations in 2021 to understand the financial implications for future LF-MDA efforts. The study looked at the cost data from LF-MDA activities conducted by the Neglected Tropical Diseases (NTD) programme of the Ghana Health Service and the SENTINEL study in the Ahanta West district for the E&T and T&T interventions. Population data from the 2021 Ghana Population and Housing Census were used to estimate the LF-MDA-eligible population in the country. The study found that implementing these strategies, either through E&T or T&T, to improve coverage comes with an additional cost to the LF elimination goals. For example, in 2024, the projected cost per person treated by the routine LF-MDA alone is estimated at US$0.83. However, when combining LF-MDA with E&T or T&T, the costs increase to US$1.62, US$2.88, respectively, for the same year. Despite the higher costs, the combined LF-MDA and mop-up strategies are estimated to treat a larger population in 2024 (1,392,211) compared to the routine LF-MDA approach (988,470) for the same year. In summary, although the combined LF-MDA and mop-up strategies have higher costs, they may offer NTD Programmes the option to improve treatment coverage and achieve the LF elimination target sooner. This is particularly important as the routine LF-MDA alone approach has been in place for many years, with some districts still facing challenges in reaching elimination targets.

## Introduction

Ghana has implemented the Global Programme to Eliminate Lymphatic Filariasis (GPELF) since June 2001 [1], covering all the endemic districts by 2006 [2]. Since the start the Ghana NTD programme has conducted the WHO-recommended Mass Drug Administration (MDA) [1] in endemic districts using a drug combination of ivermectin and albendazole. By 2016, 81 of the 98 initial endemic districts had reached a microfilaria (mf) prevalence <1%, or an antigen (Ag) prevalence <2%, had passed the Transmission Assessment Survey (TAS) and stopped MDA. The remaining districts failed pre-TAS and still had mf prevalence >1% despite several years of LF-MDA programme, exceeding the recommended 6 annual rounds of MDA by WHO, partly due to relatively high baseline mf prevalence levels [3]. As of 2021, the Neglected Tropical Disease (NTD) programme reported 11 remaining districts still undertaking MDA with 15 to 19 years of MDAs. In these districts, many challenges have been reported, including individuals who seldom or never take part in MDA. This poses a threat to the success of the programme, as they may serve as reservoirs of infection, re-infecting their communities. The MDA in Ghana relies on the use of registers that detail the names, ages, drugs received, and the reasons for non-treatment. Thus, the LF SENTINEL study was undertaken in 2021 to develop and implement two mop-up strategies namely, Engage and Treat (E&T) and Test and Treat (T&T) strategies to reach persons who did not receive MDA in the endemic communities in the Ahanta West District of Ghana [4].

In the E&T strategy, all individuals who missed the last MDA were identified from the community treatment registers, approached and engaged to receive treatment. If they agreed, they were given the MDA drugs ivermectin and albendazole following the treatment guidelines of the NTD programme. However, if they refused, they were considered for the T&T strategy and offered testing to determine whether they have the infection. If positive, they were further engaged to receive treatment. The major difference between the E&T and T&T is that the E&T individuals usually miss MDA due to sickness, absence, or travel, while the T&T individuals typically refuse MDA due to fear of adverse events or low-risk perceptions. The E&T was mostly implemented by the Community Drug Distributors (CDDs), while the T&T was implemented by the Community Health Nurses (CHNs) who already perform malaria testing based on the rapid diagnostics tests and were trained to perform testing for LF using the Filaria Test Strip (FTS) [4]. Both the CDDs and CHNs were incentivized for these activities since they are undertaken after the routine MDA programme. The LF SENTINEL study reached an additional 23,879 individuals after the LF-MDA, 95.61% of whom were treated. Further, an FTS positivity of 8.66% was observed among the T&T population [4].

Most studies on the economic evaluation of MDAs for various diseases show that they are cost-effective strategies. These studies are LF-MDA in the Philippines [5]; LF-MDA in Haiti [6]; trachoma MDA in South Sudan [7]; systematic review of economic evaluations of LF interventions [8]; overview of the MDA programme [9]; scabies MDA in Fiji [10]; malaria MDA modelling [11]; schistosomiasis MDA in South Africa [12]; focal malaria prevention MDA in Zambia [13]; programmatic cost of targeted mass drug administration for malaria in Myanmar [14]; and assessment of the health economic studies of Global Programme to Eliminate Lymphatic Filariasis (GPELF) [15]. Thus, the current study sought to answer the following questions: (1) What is the financial cost of the LF-MDA in Ghana? (2) What is the financial cost of the E&T/T&T implementation approaches? and (3) Comparatively, is it financially worth implementing the LF-MDA in its current state alone or in combination with the E&T/T&T implementation approaches? These estimates are important to inform future cost-effectiveness studies of LF MDA in Ghana and other LF-endemic countries.

## Methods

### Ethics statement

Ethical approval for the SENTINEL study was obtained from the Noguchi Memorial Institute for Medical Research (NMIMR) Institutional Review Board (CPN 021/19-20 *amend*. *2022*) with Federal Wide Assurance Registration (FWA 00001824). However, for this cost analysis study, no data was directly collected from study participants. As such, consent from participants for the publication of this paper is not applicable.

### Study protocol

This costing study covers the 2021 LF-MDA implementation activities carried out by the Ghana NTD programme. In 2021, the LF-MDA activities were conducted in eleven (11) districts within five (5) regions in Ghana, namely: Sunyani Municipal and Sunyani West district in the Bono region; Bole and Sawla-Tuna-Kalba districts in the Savanah region; Nabdam district in the Upper East region; Lawra, Wa-West and Wa-East districts in the Upper West Region and the Ahanta West, Ellembelle and Nzema-East districts of the Western region. The MDA campaign was carried out from 15th to 28th April 2021.

*Perspective*: The analysis was conducted from the perspective of the NTD Programme. This is a cross-sectional costing study using the ingredient method approach [16]. It involved retrospective analysis of the cost data of the LF-MDA activities. The LF SENTINEL [4] study data analysis involved retrospective cost data of the two interventions (i.e., E&T and T&T).

### Study area and level of analysis

All 11 participating districts in 2021 were used for the MDA cost analysis. The costing exercise gathered information from the NTD programme. According to the NTD Programme, all 11 districts as of 2021 had treatment coverage rates above 80% [17] in each district (Table 1). From 2013–2019, the reported average coverage rate of the annual LF-MDA by the Ghana NTD programme is 86.5%. In 2021, the Ghana NTD Programme reported that LF-MDA in these 11 districts treated 753,219 people, resulting in a reported coverage rate of 85.6% as shown in Table 1.

**Table 1:** LF endemic district population and LF-MDA coverage reported by the NTD programme (2021). * = ICT prevalence: & = mf prevalence. Baseline prevalence averaged from the mapping data available on the ESPEN website.

Regions	Districts	Baseline prevalence	No. of Communities covered by 2021 LF-MDA	Population treated by LF-MDA	Previous LF-MDAs conducted as of 2021	NTD Programme reported coverage for 2021 (%)
**Bono**	**Sunyani Municipal**	21.0*	139	94,014	15	83.1
	**Sunyani West**	21.0*	106	72,836	15	86.9
**Savannah**	**Bole**	4.6*	197	62,018	16	81.7
	**Sawla-Tuna-Kalba**	5.4*	306	86,957	15	86.5
**Upper East**	**Nabdam**	8.0*	84	35,774	18	87.8
**Upper West**	**Lawra**	80.5*	123	43,487	18	88.4
	**Wa West**	4.4*	225	62,212	18	83.9
	**Wa East**	32.0*	153	62,212	18	83.9
**Western**	**Ahanta West**	41.8*	116	103,145	19	88.5
	**Ellembelle**	18.0*	103	68,516	18	84.3
	**Nzema East**	**1.5** ^**&**^	108	62,048	16	86.4
	**Total**		**1,660**	**753,219**	**186**	**85.6**

### Financial cost analysis

**Fig 1:**
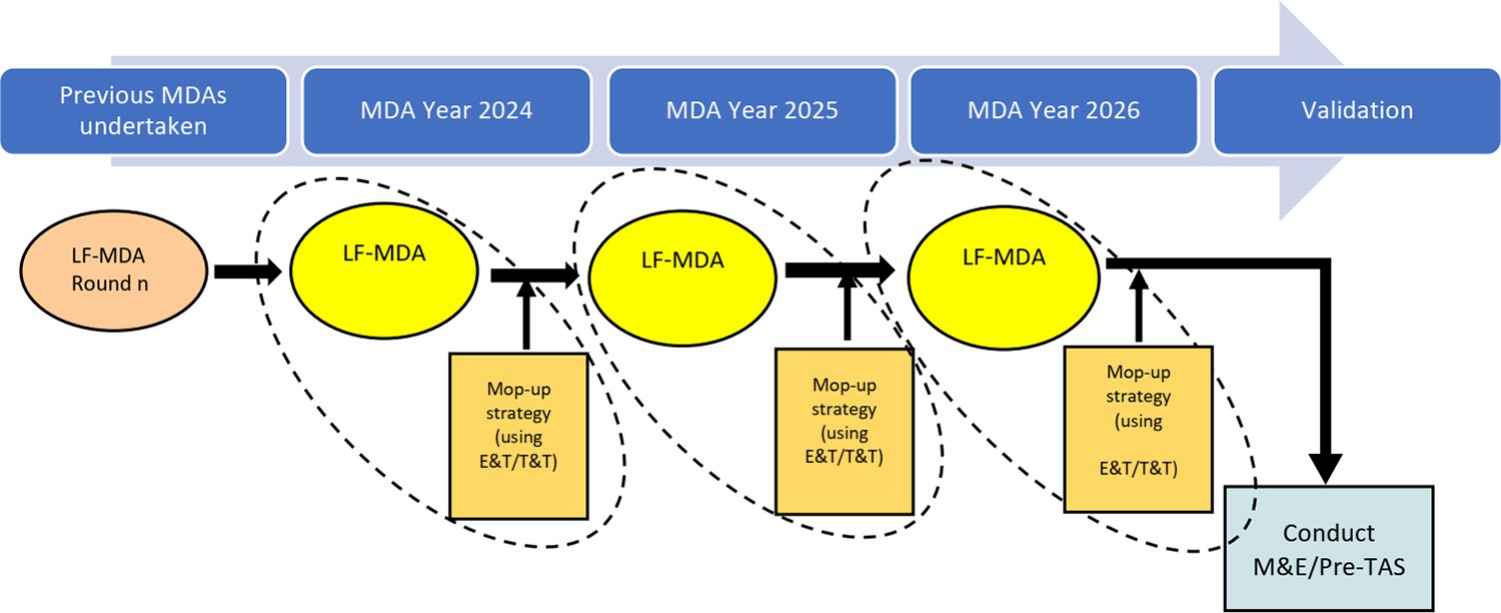
Proposed LF-MDA implementation (Years 2024, 2025 and 2026) with mop-up strategies (E&T/T&T) in settings with persistent transmission.

There were two main financial analyses undertaken, an estimation of the financial cost of LF-MDA and the estimation of the financial cost for a proposed LF-MDA supplemented with mop-up strategies i.e., E&T/T&T. For the analysis, each year of MDA implementation (i.e., 2024, 2025 and 2026) was considered a package of LF-MDA and a mop-up strategy i.e., E&T/T&T. For the three years (2024–2026), the cost of LF-MDA + E&T + T&T was estimated. Although M&E assessments (i.e., pre-TAS and TAS) are carried out as part of the LF-MDA implementation process for validation purposes, these costs were not considered. The cost of restarting the proposed interventions was also not considered. Based on data from the LF SENTINEL study [4], the routine LF-MDA’s reported coverage was adjusted to 71%. This coverage rate was projected and treated populations were estimated. Fig 1 shows the diagrammatical presentation of the proposed LF-MDA implementation with mop-up strategies (E&T/T&T) in settings with persistent transmission.

#### Data collection

Administrators and finance staff of the NTD Programme provided retrospective LF-MDA cost data [18]. Only costs related to the LF-MDA activities were collected and categorised [16, 19]. Researchers of the LF SENTINEL study [4] in Ahanta West also provided retrospective cost data on the study. The cost categories of the LF-MDA activities include MDA Launch, social mobilization, training, personnel (all salary proportions and allowances of personnel involved in the LF-MDA implementation), monitoring and supervision, drugs and other supplies distribution, further grouped into recurrent and capital costs for analysis (S1 Table). LF-MDA activities were further regrouped into national, regional and district levels activities. All drugs used for the MDA campaign were donated by the WHO [20], thus drug costs were not considered in the financial cost analysis. Adverse event treatment cost was not considered in this study because no cases were reported.

#### Data analysis

All the analysis was carried out using Microsoft Excel software. The cost data were in Ghana cedis (GHS) and were converted to U.S. dollars (US$). The annual average Bank of Ghana (BOG) exchange rate was used for 2021 to convert costs in Ghana cedis to their US dollar equivalents (i.e., US$1 = GHS5.82). Projected costs for 2022–2026 were done using the 2018–2022 annual average Bank of Ghana (BOG) exchange rate (i.e., US$1 = GHS5.95) at the time of the analysis [21]. Cost results are presented in tables and charts.

### Estimating the eligible, treated and untreated population

The population for the LF-MDA was estimated using data from the Ghana 2021 Population and Housing Census (PHC) conducted by the Ghana Statistical Service. The census-published statistics gave no age distribution by district; however, the regional age distribution shows that the under-five years population constitutes about 3.5–6% of the total regional population [22]. Hence the district populations were adjusted to remove the under-five years population using the regional estimates. The regional population growth rates were used to estimate the eligible LF-MDA population including the under-five years population who will attain LF-MDA eligible age for each projected year. The average adult crude mortality rate for 2019–2022 (7.4 per 1,000 population) [23] was also used to adjust the projected populations from 2024–2026 (S4 Table).

#### Estimating the costs of LF-MDA for 2021 based on programme data

Capital costs, defined as items with a useful life of more than one year, were collected for transport (vehicles) and office equipment (laptops, printers, projectors, scanners, tablets and pointers). Replacement costs of the capital items were used in the analysis. The financial capital cost was obtained by dividing the cost of the capital item by its useful life. Recurrent costs were calculated from the national, regional and district levels (S2 Table). The total costs of the national, regional and district levels activities were obtained by summing all the individual costs of these activities incurred by the LF-MDA programme. All LF-MDA category’s total costs were shared equally among the 11 districts covered by the 2021 LF-MDA. The NTD Programme financial cost per person treated for the 2021 LF-MDA was used in calculating the total financial cost of the LF-MDA using the 2021 PHC-eligible LF-MDA population. The average cost per person treated was calculated by dividing the total cost of the LF-MDA in 2021 by the reported number of people treated by the NTD Programme.

#### Estimating the costs of LF-MDA and E&T/T&T for the period 2024–2026

The 2024–2026 projected MDA financial costs were estimated by adjusting the 2021 LF-MDA financial cost for inflation. The financial costs were adjusted using the 2022 average annual inflation rate of 31.47% [24] and the five-year average annual inflation rate of 13.9% (2018–2022). This was done to establish the financial cost for subsequent LF-MDAs and to make our cost projections stable in the medium term. The LF SENTINEL Study [4] found double the untreated population reported by the NTD Programme in Ahanta West, therefore we doubled the untreated population in each district from a national average of 14.4% to 29% in this study.

#### Estimating the costs of E&T/T&T based on data from the SENTINEL study for Ahanta West and all 11 districts

The cost of the E&T and T&T were estimated as follows. Replacement costs of the capital items were used. The financial capital cost was obtained by dividing the cost of the capital item by its useful life. Recurrent cost items in the E&T and T&T were made up of the following: Planning, Supplies and accessories, Training of CDDs and Community Health Nurses (i.e., CHN), Community Mobilisation, CDD Personnel Allowance (E&T), and CHN Personnel allowance (T&T). The cost of Monitoring and Evaluation by study personnel was not included, as it was deemed to be mainly a research cost. The total costs for the capital and recurrent costs were then shared between the mop-up strategies (i.e., E&T and T&T) using the proportion of personnel trained for each strategy (i.e., CDDs for E&T (80%) and CHNs for T&T (20%)) respectively. The shared recurrent costs of the mop-up strategies were further apportioned between the two interventions (i.e., E&T and T&T) also using the proportion of personnel trained (i.e., CDDs for E&T (80%) and CHNs for T&T (20%)) respectively. Drugs used by the mop-up strategies were also donated and thus were not included in the financial cost analysis. The analysis of the T&T strategy considered two approaches: (1) an NTD-Programme-Led T&T mop-up strategy, implemented directly by the NTD programme by deploying officers into the communities to test and treat the population; this was estimated to cost US$13.08 per person treated; and (2) a health system integrated T&T mop-up strategy (i.e., the T&T strategy is adapted into Ghana’s health system and becomes part of CHNs day-to-day activities), implemented by the CHNs in the Community-based Health Planning Service (CHPS) zones in the communities within the districts. The T&T mop-up strategy integrated into the health system was further costed in two different categories: (1) without any allowances paid since T&T will be part of CHN daily activities; and (2) the NTD Programme paying CHNs the same per diems as the CDDs. These different approaches in costing the T&T (although beyond what was done in the SENTINEL study) were assessed to provide options for adoption by national programmes. Both the E&T and T&T strategies were costed based on a 14-day implementation period. Further cost assumptions are presented in the S3 Table.

### Sensitivity analysis

After costs were projected using the five-year annual inflation rate of 13.9% in carrying out our financial analysis, one-way sensitivity analyses were conducted to explore the effects of key assumptions on the cost estimate. The effects of varying the following parameters were explored on the projected costs for the sensitivity analysis: i) the useful life of vehicles was increased to 7.5 years [7], and ii) the exchange rate was modified from the five-year (i.e., 2018–2022) annual average of GHS5.95 per US$ by Bank of Ghana to that provided by OANDA [25] at an average of GHS6.11 per USD for the same period.

## Results

### Projected eligible LF-MDA population for 2024–2026

From the 2021 Population and Housing Census (PHC), the total population in the 11 districts was 1,225,443 [26]. The total eligible LF-MDA population in 2021 for the 11 districts was estimated to be 1,172,360. However, the NTD Programme reported reaching 753,219 people (Table 1). Table 2 shows the projected total eligible LF-MDA populations for subsequent years based on the estimated eligible population. Using the adjusted routine LF-MDA coverage of 71%, the treated populations were estimated. The estimated untreated population of 29% and E&T and T&T coverage of the estimated untreated population are also shown in Table 2. Further details are in the S4–S6 Tables.

**Table 2:** Projected eligible LF-MDA population for 2024–2026.

Years	Total Estimated LF-MDA Eligible Population	Adjusted NTD programme reported Coverage (71%) ^b^	Estimated untreated population (29%) ^c^	Estimated E&T coverage of 75.9% untreated population	Estimated T&T coverage of 24.1% of the untreated population
**2024**	1,392,211	988,470	403,741	306,440	97,302
**2025**	1,474,647	1,047,000	427,648	324,585	103,063
**2026**	1,562,149	1,109,126	453,023	343,845	109,179

^**a**^ = Total eligible population to be covered by LF-MDA according to population and housing census

^**b**^ = NTD programme coverage rate of 71% based on NMIMR data

^**c**^ = Estimated untreated population based on NMIMR data

^**d**^ = Estimated E&T coverage rate based on NMIMR

^**e**^ = Estimated T&T coverage rate based on NMIMR data.

### The financial cost of Ghana’s LF-MDA in 2021

The total financial cost of the NTD Programme LF-MDA for 2021 was US$377,715.50 of which 65% was recurrent cost. About 41% of the cost was at the district level. The most substantial inputs of the 2021 LF-MDA went into training, personnel and supervision, and monitoring costs, as shown in Table 3.

**Table 3:** Financial cost of Ghana’s 2021 LF-MDA in US$.

Recurrent Items:	
National:	Financial cost (%)
MDA Launch	5,111.68 (1.4)
Training of Trainers Meeting	22,362.54 (5.9)
District Training Support	8,505.15 (2.3)
Supervision & Monitoring (Technical)	18,608.25 (4.9)
Items for CDDs & Health Workers	19,020.62 (5.0)
National NTD Programme Staff	1,669.12 (0.4)
Sub-total:	75,277.37 (19.0)
**Regional:**	
Regional Social Mobilization	859.11 (0.2)
Regional Training Support to Districts [per district]	4,965.64 (1.3)
Regional Monitoring Support to Districts [per district]	10,567.01 (2.8)
Regional NTD Programme Staff	589.10 (0.2)
Sub-total:	16,980.85 (4.5)
**District:**	
Training of CDDs	35,510.31 (9.4)
District Level training [per sub-district]	5,500.00 (1.5)
District Social Mobilization	9,091.07 (2.4)
MDA Implementation by CDD	77,598.45 (20.5)
Sub-district Level Monitoring (per sub-district)	16,301.55 (4.3)
District Level Monitoring	9,106.53 (2.4)
Sub-total:	153,107.90 (40.5)
Total recurrent cost:	245,366.13 (65.0)
**Capital Items:**	
Vehicles	118,762.89 (31.4)
Office Equipment	13,586.49 (3.6)
Sub-total:	132,349.37 (35.0)
Total	377,715.50 (100.0)

### Cost per person treated in LF-MDA for 2021

**Fig 2:**
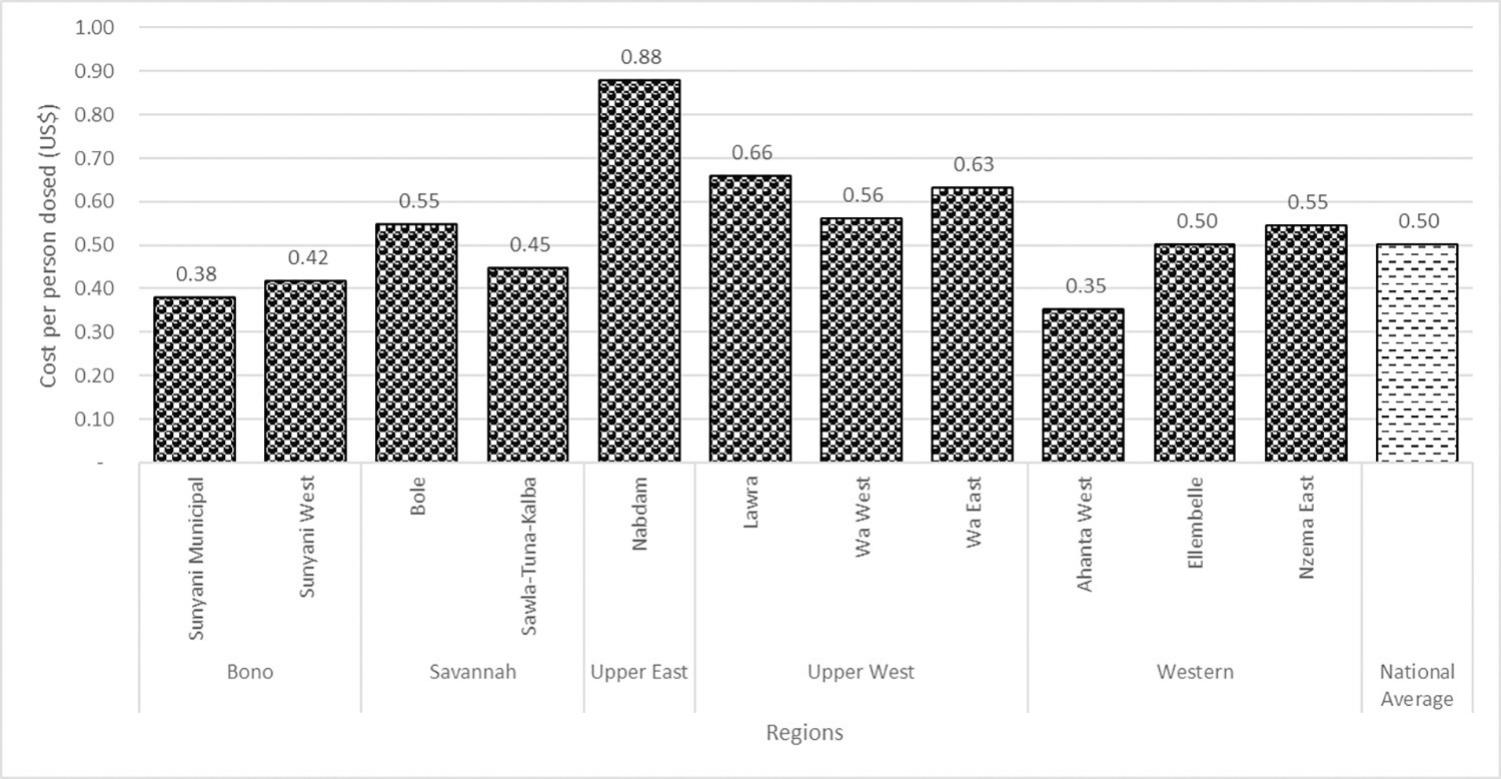
Financial cost per person treated in the 2021 LF-MDA.

Fig 2 shows the financial cost per person treated in the 2021 LF-MDA. The national average financial cost per person treated was estimated to be US$0.50 based on the reported number of persons treated by the NTD Programme.

### Financial cost of Engage and Treat (E&T) and Test and Treat (T&T) SENTINEL study in Ahanta West district

In Ahanta West district, the SENTINEL study reached 26,394 untreated people [4]. 75.9% of this untreated population was treated through E&T, while the remaining 24.1% were targeted by T&T. E&T was estimated to cost US$53,569.52 while T&T was estimated to cost US$32,409.17. The main E&T cost driver was CDD allowances (i.e., 12.8%), while that for T&T were supplies and accessories (i.e., 46.1%) and CHN allowances (i.e., 16.8%) as shown in Table 4. The study estimated E&T cost per person treated to be US$2.68 and for T&T to be US$5.10. The investigators incentivised CDDs at a higher rate than the NTD programme. CDDs were paid an allowance of approximately US$0.34 per person treated for E&T while CHNs were paid US$0.86 per person treated for T&T.

**Table 4:** Financial cost of E&T and T&T implementation by NMIMR in Ahanta West district (US$).

	Engage and Treat (E&T)	Test and Treat (T&T)
	Cost (%)	Cost (%)
**Planning**		
Transport Cost	2,004.54 (3.7)	501.13 (1.5)
Photocopy	354.50 (0.7)	88.63 (0.3)
Stationery	38.63 (0.1)	9.66 (0.0)
Researchers Allowance	3,450.17 (6.4)	862.54 (2.7)
***Sub-Total***	5,847.84 (10.9)	1,461.96 (4.5)
**Supplies and accessories**		
Test Kits	-	13,420.23 (41.4)
Lancet and Vacutainers	-	329.90 (1.0)
Filter Paper	-	592.70 (1.8)
Nose Masks	-	395.19 (1.2)
Alcohol Swabs	-	193.30 (0.6)
***Sub-Total***	-	14,931.31 (46.1)
**Training of CDDs and CHNs**		
Venue hiring	82.47 (0.2)	20.62 (0.1)
Transport and Lunch for participants	618.56 (1.2)	154.64 (0.5)
Printing Training Manual, Data Sheet, and Referral Forms	57.73 (0.1)	14.43 (0.0)
Lancets	-	206.19 (0.6)
Vacutainers	-	144.33 (0.4)
Facemasks for Frontline Health workers	247.42 (0.5)	61.86 (0.2)
Allowance for 2 Disease Control Officers	41.24 (0.1)	10.31 (0.0)
Transport Cost	412.37 (0.8)	103.09 (0.3)
Research Assistants Allowance	412.37 (0.8)	103.09 (0.3)
Driver Allowance	206.19 (0.4)	51.55 (0.2)
Research Fellow Allowance	577.32 (1.1)	144.33 (0.4)
Miscellaneous	146.80 (0.3)	36.70 (0.1)
***Sub-Total***	2,802.47 (5.2)	1,051.13 (3.2)
**Community Mobilisation**		
Translation from English to Ahanta	20.62 (0.0)	5.15 (0.0)
**Personnel Allowance**		
CDD allowance	6,878.01 (12.8)	-
CHN Allowance	-	5,454.47 (16.8)
**Capital Cost**		
Vehicles	35,738.83 (66.7)	8,934.71 (27.6)
Office Equipment	2,281.75 (4.3)	570.44 (1.8)
***Sub-Total***	38,020.58 (71.0)	9,505.15 (29.3)
**Total**	**53,569.52 (100.0)**	**32,409.17 (100.0)**

### The projected financial cost for the routine LF-MDA using the PHC LF-MDA eligible population

**Fig 3:**
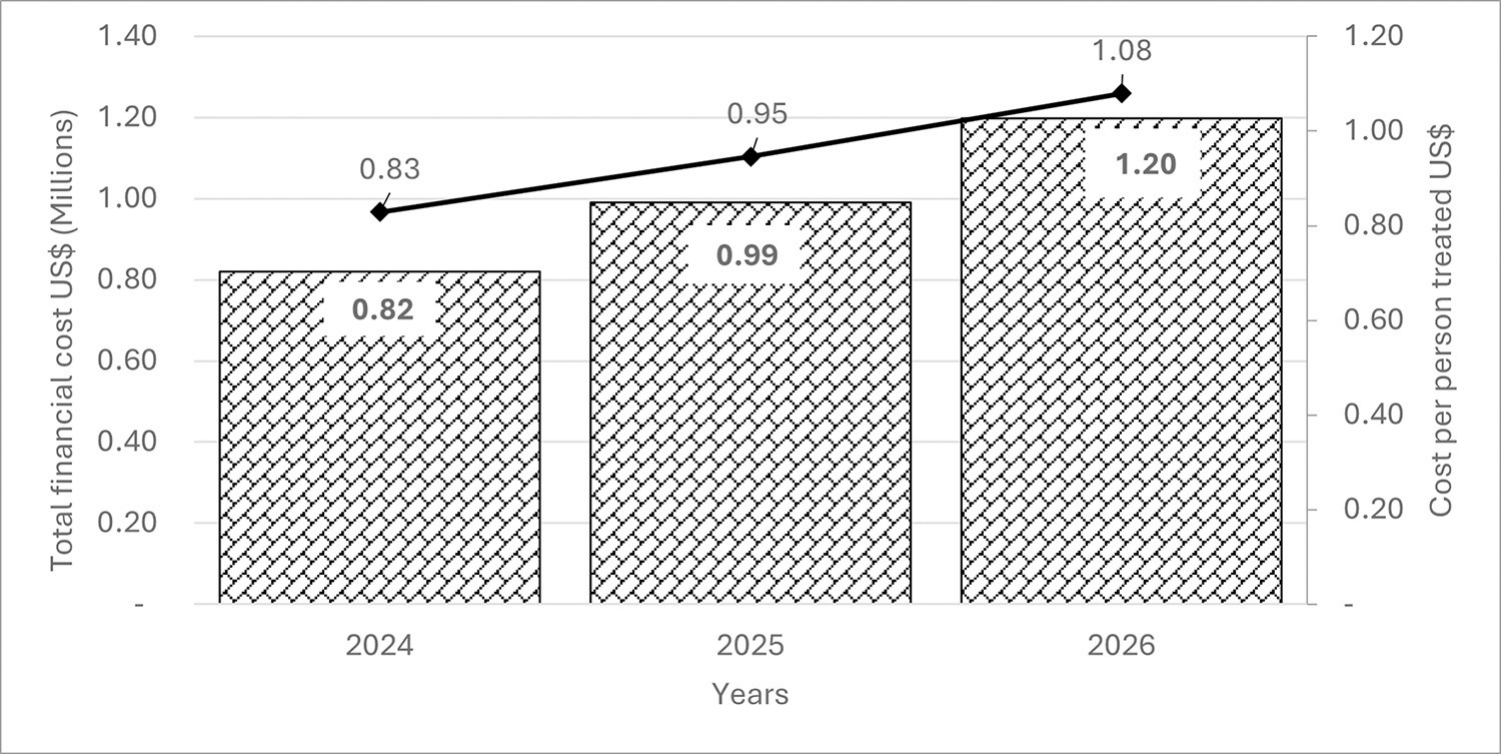
Projected financial costs and costs per person treated in the routine LF-MDA from 2024 to 2026. The bars represent the projected financial costs, while the line represents the cost per person.

Using a multi-year average exchange rate for projections accounts for currency fluctuations [27], Fig 3 shows the projected financial costs and cost per person treated in the routine LF-MDA with the adjusted coverage rate of 71% as projected from 2024 to 2026 for the LF-MDA eligible population. Detailed district breakdown are shown in S7 and S8 Tables.

### Estimated and projected financial cost of E&T/T&T mop-up strategies

**Fig 4:**
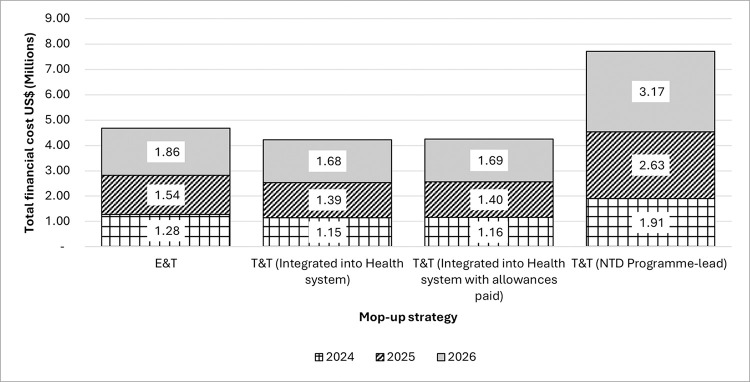
Projected total financial costs of the E&T and T&T strategies from 2024 to 2026.

Cost per person treated using E&T and T&T from Ahanta West were extrapolated to the other ten districts. Estimating the E&T mop-up strategy cost per person treated using the NTD Programme per diem for CDDs amounts to US$2.44 per person treated. The estimated cost per person treated using the T&T mop-up strategy integrated into the health system with no allowances paid to CHNs was US$6.93. With allowances paid the cost per person was US$6.98. Fig 4 shows the total financial costs of the E&T and T&T strategies (i.e., NTD-programme-led, integrated into the health system either with or without allowances paid) implemented in three annual cycles from 2024–2026 (see S9–S11 Tables for detailed breakdown). The five-year average annual inflation rate of 13.9% (2018–2022) [24] was also used in adjusting the costs.

### Estimated cost of proposed combined LF-MDA and E&T/T&T mop-up strategies

**Fig 5:**
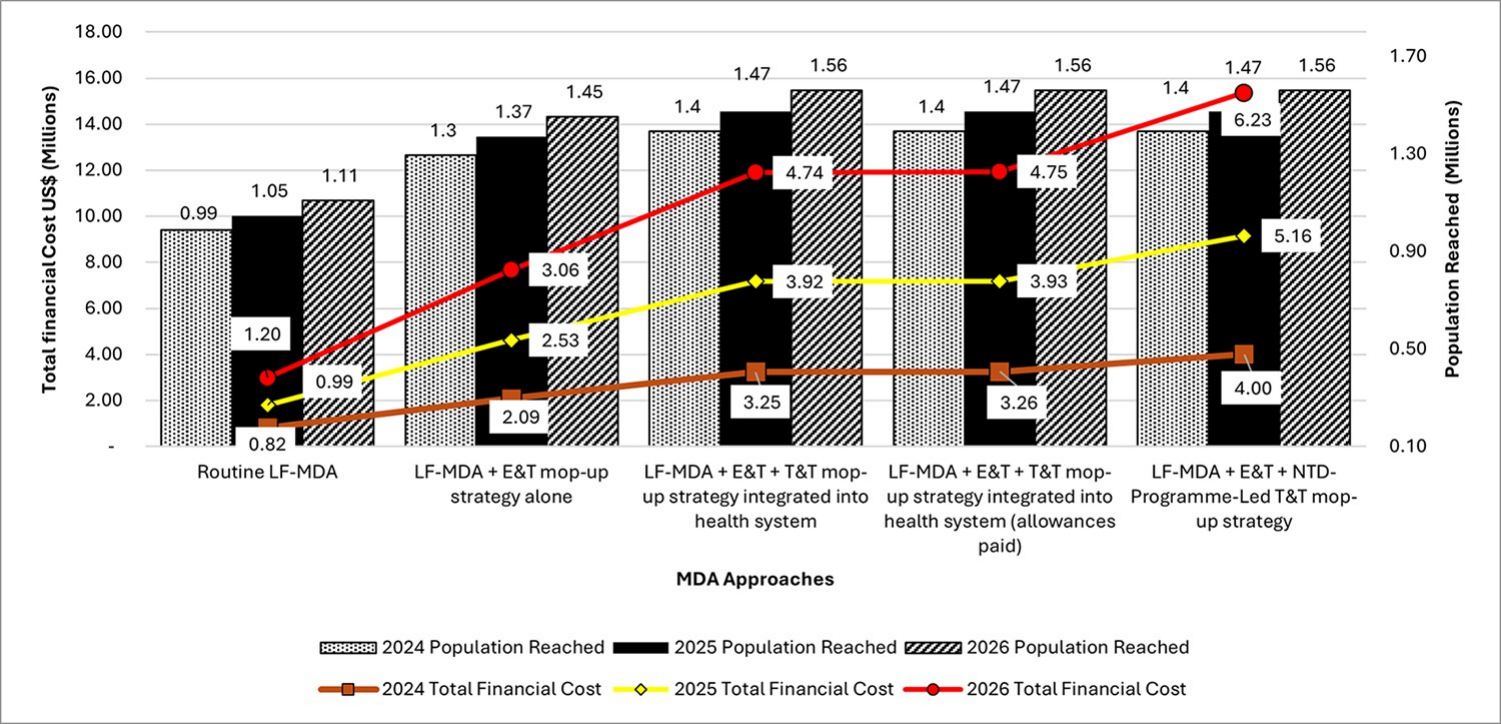
Comparison between the projected LF-MDA eligible population treated and their total financial costs from 2024 to 2026. The bars represent the population reached and the lines represent the total financial cost.

**Fig 6:**
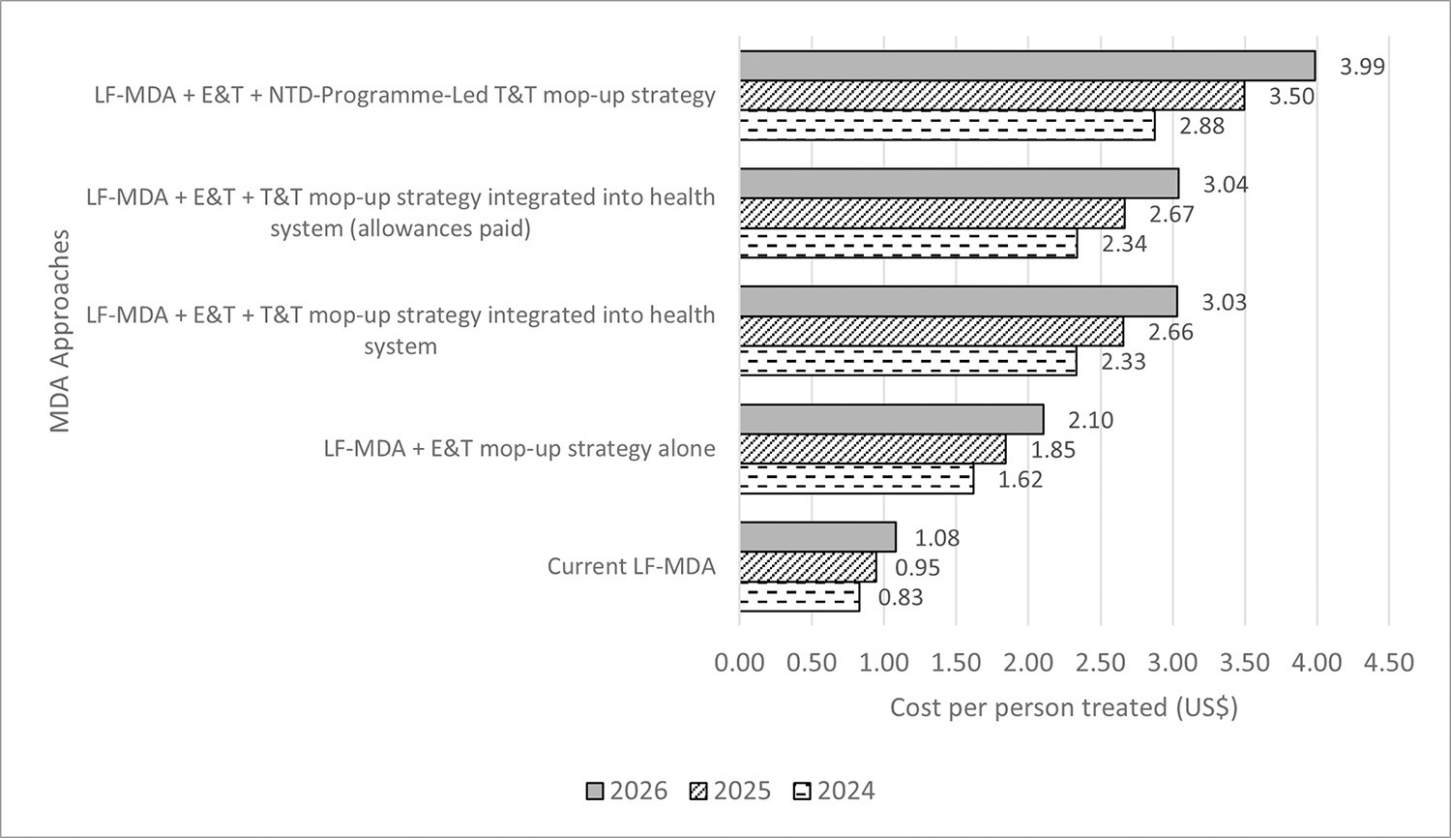
Projected cost per person per treatment approach.

Fig 5 shows the comparison between the projected LF-MDA eligible population treated using the routine LF-MDA and the proposed LF-MDA with mop-up strategies. The figure also shows the comparison of the estimated total financial cost of the routine LF-MDA and the proposed LF-MDA and E&T/T&T mop-up strategies projected in three annual cycles from 2024–2026. Fig 6 shows the cost per person treated per approach over the same period. The proposed combined strategies have a higher cost compared to the routine LF-MDA, due to the added component of the mop-up strategy. However, the proposed combined LF-MDA and mop-up strategies have a higher estimated treated population (i.e., 40% more) compared to the routine LF-MDA approach (S12 Table).

### Sensitivity analysis

After projecting costs using the five-year annual inflation rate of 13.9%, the effect of the sensitivity analysis carried out showed the following: the effect of increasing vehicle useful life from 5 years to 7.5 years had the most pronounced effect by reducing cost per person by ranging from 12.2 to 14.5% for 2024; 11.1 to 14.6% for 2025 and 5.6 to 11.3% in 2026 shown in Table 5. Modifying the exchange rate from the Bank of Ghana’s average annual five-year rate (i.e., 2018–2022) of GHS5.95 to the OANDA GHS6.11 during the same period showed an average reduction in cost per person treated ranging from 0.6 to 2.7% for 2024; 1.0 to 3.3% in 2025; and 0.9 to 2.9% in 2026 shown in Table 6.

**Table 5:** Sensitivity analysis results of US$/per person treated with a change in vehicle useful life*.

S/n	Approach	2024			2025			2026		
		Base case	Sensitivity analysis estimate	% deviation from Base case	Base case	Sensitivity analysis estimate	% deviation from Base case	Base case	Sensitivity analysis estimate	% deviation from Base case
1	**Routine LF-MDA**	0.83	**0.74**	-12.2	0.95	**0.85**	-11.8	1.08	**0.97**	-11.3
2	**LF-MDA + E&T mop-up strategy alone**	1.62	**1.42**	-12.3	1.85	**1.62**	-12.4	2.11	**1.85**	-5.6
3	**LF-MDA + E&T + NTD-Programme-Led T&T mop-up strategy**	2.88	**2.55**	-11.4	3.50	**3.11**	-11.1	3.99	**3.54**	-1.6
4	**LF-MDA + E&T + T&T mop-up strategy integrated into health system**	2.33	**2.00**	-14.1	2.66	**2.28**	-14.2	3.03	**2.59**	-6.5
5	**LF-MDA + E&T + T&T mop-up strategy integrated into the health system (allowances paid)**	2.34	**2.00**	-14.5	2.67	**2.28**	-14.6	3.04	**2.59**	-6.8

*Effect of increasing vehicle useful life from 5 years to 7.5 years.

**Table 6:** Sensitivity analysis results of US$/per person treated with a change in exchange rate*.

S/n	Approach	2024			2025			2026		
		Base case	Sensitivity analysis estimate	% deviation from Base case	Base case	Sensitivity analysis estimate	% deviation from Base case	Base case	Sensitivity analysis estimate	% deviation from Base case
1	**Routine LF-MDA**	0.83	**0.81**	-2.5	0.95	**0.92**	-3.3	1.08	**1.05**	-2.9
2	**LF-MDA + E&T mop-up strategy alone**	1.62	**1.61**	-0.6	1.85	**1.83**	-1.0	2.11	**2.09**	-0.9
3	**LF-MDA + E&T + NTD-Programme-Led T&T mop-up strategy**	2.88	**2.80**	-2.7	3.50	**3.40**	-2.8	3.99	**3.88**	-2.8
4	**LF-MDA + E&T + T&T mop-up strategy integrated into health system**	2.33	**2.28**	-2.1	2.66	**2.60**	-2.3	3.03	**2.97**	-2.0
5	**LF-MDA + E&T + T&T mop-up strategy integrated into the health system (allowances paid)**	2.34	**2.29**	-2.1	2.67	**2.61**	-2.2	3.04	**2.98**	-2.0

*Effect of adjusting five-year average annual exchange rate from US$1 = GHS5.95 given by the Bank of Ghana to US$1 = GHS6.11 as provided by OANDA

## Discussion

There are currently many endgame challenges to the global LF elimination programme. Addressing these challenges is therefore critical if the tremendous gains made are to be sustained. Turner (2020) recognized the need for changes to the current approach to reach elimination targets set by the WHO [15]. While different strategies for sustainability are considered, understanding the cost implication for these strategies is important to inform the resource requirements. In this context, this study assessed the cost of implementing E&T and T&T strategies [4] as part of the MDA activities.

Gyapong et al. [9] have expressed *“the need to accelerate MDA strategies to reduce the time required to interrupt transmission to meet the 2020 target for elimination of LF as a public health problem”*. However, this will depend on funding. As Turner (2020) noted, estimates of the cost of MDA are a vital component for subsequent economic evaluation of country programmes [15], this study is filling the cost studies gap in Ghana. As other studies have further observed, interventions such as MDA can have strong economies of scale (i.e., as the number of people treated increases, the cost per treatment decreases) [28]. Such financial cost estimate of the national MDA programme is critical and vital economic information for countries to advocate for increasing resources, guide budgeting and benchmark accountability. Goldman et al. [29] also indicated that *“such findings can be used on a national scale for programme planning*, *development*, *and fundraising*, *and on a global scale for calculating current global costs*, *predicting scale-up costs*, *and calculating savings from integration with other programmes*. *These results also will form the basis for guiding cost-effectiveness and cost-benefit analyses*, *as more information on the effectiveness of MDAs in the study countries becomes available”*.

In this study, we estimated the LF-MDA eligible population to be 1,172,360 in 2021 using PHC data, however, we know from previous studies that issues of data accuracy are common in reported coverage estimates [9, 30]. This is further buttressed by the study that found that 60% of data recorded in some endemic districts in Ghana were inaccurate [31]. In 2021, the NTD Programme reported a treatment coverage of 85.6% after treating 753,219 people in the eleven endemic districts [17], with 103,145 people treated in Ahanta West district alone. The NTD Programme reported an estimated 12% (i.e., 13,422) of the district’s eligible population were untreated. However, the LF SENTINEL study discovered double the number of untreated people (i.e., 26,394), 78% of them not captured in the MDA register for the district [4]. The proposed LF-MDA and mop-up approaches have a 40% larger projected treated population than the present MDA approach (Fig 6).

This study provides the projected routine LF-MDA financial costs for 2024–2026. The routine implementation cost to Ghana’s LF-MDA will range between US$819,504.54 (2024) to US$1,197,513.11 (2026), assuming MDA is still implemented in all 11 districts. The proposed LF-MDA with mop-up strategies namely: LF-MDA + E&T alone; LF-MDA + E&T + NTD-Programme-Lead T&T having the highest cost due to added administrative and personnel cost by NTD Programme officers; LF-MDA + E&T + T&T integrated into health system; and LF-MDA + E&T + T&T integrated into health system (allowances paid) are all higher in total cost with the added strategy which treats a higher population leading to more persons treated. Total financial costs range from US$2,094,897.49 (2024) to US$6,228,991.11 (2026). Goldman and colleagues [29] estimated Ghana’s 2002 financial cost for implementing the LF-MDA at US$1,358,000 (i.e., about US$2,307,550.74 when inflation-adjusted).

The routine LF-MDA in 2021, had an estimated cost per person treated of US$0.50. The projected estimated cost per person treated for 2024, 2025 and 2026 are US$0.83, US$0.95, and US$1.08 respectively. The proposed LF-MDA combined with the additional strategies have estimated costs per person treated ranging from US$1.62 to US$3.99 for the same period. Goldman et al, (2007) [29] estimated country MDA costs to be between US$0.06 to US$2.23. Similar studies found the cost per person treated in Haiti’s LF-MDA to be US$0.42 [6]. Others showed an average financial cost of MDA to be US$0.46 per person treated [8]. Sudan’s trachoma MDA was calculated to be US$1.53 per person treated [7]. The programmatic cost of targeted MDA in Myanmar was estimated to be US$2.50 per person treated [14]. Thus, in comparison to other MDA costing studies, our financial cost per person estimates are well within the range of previously reported MDA studies. With the added advantage of reaching a much larger population for the LF-MDA, combining LF-MDA with E&T/T&T will serve to speed up LF elimination. Studies have shown that MDA programme costs can be higher when it aims to eliminate transmission compared to when its aim is only morbidity control [8].

This MDA programme cost estimates are high as shown in studies from countries in the AFRO region and South Africa [32, 13]. The implication is that endemic countries will require the greatest investment to reach elimination or eradication, but also stand to gain the most in cost savings. In light of this, a substantial national government budget and technical assistance will be required. Thus, to sustain the MDA programme over time until elimination is achieved, policymakers must therefore consider a trade-off between investing fully in the proven elimination strategies now or in bits and pieces. As the situation is currently this might not sustain the programme to achieve its goal. Secondly, donor support in terms of drug donation and increased funding is crucial to the programme. Hence governments should reinvigorate the partnership with the WHO, the Mectizan Donation Program and other donors who support the LF-MDA programme. However, studies have observed successfully eradicating LF depends on more than monetary investment. Political will, continued community ownership, and the feasibility of the campaign all need to be considered [33].

We acknowledge that the E&T/T&T approach is much more expensive with the combination of the E&T approach with the MDA being twice the cost of the routine MDA while the further inclusion of the T&T approach increases the cost to three times the cost of regular MDA. While these costs are significant, it is worth noting that these strategies are not expected to be implemented in all areas, but rather in settings with persistent transmission, and where investigations reveal the presence of individuals who seldom or never take part in MDA. These can include districts that have failed pre-TAS or TAS or where the resurgence of infection has been demonstrated with a need to restart MDA. In these settings, the high coverage may offset the increased cost of the interventions and help attain elimination faster. In implementing these strategies, the option where the E&T and T&T combination is applied and the T&T approach integrated into the health system (with allowances paid) is recommended, considering the insignificant difference when allowances are not paid. The payment of allowances may serve as motivation to the personnel involved in the conduct of these activities [34, 35]. A well-resourced and functional CHPS zone translates to substantial health benefits with positive health and economic benefits. Therefore, governments and stakeholders need to increase investments in LF elimination to accelerate the achievement of universal health coverage (UHC) by Ghana’s roadmap of 2020–2030. This investment will pay off and it will in the long-term save the NTD programme from doing additional rounds of MDA ad nauseum, which is unreasonable given it has already taken 15+ rounds and counting in some districts. Thus, looking at the long-term implications, combining LF-MDA with additional strategies will pay off by reaching the elimination targets earlier.

We estimated the financial programmatic cost of the LF MDA programme in Ghana using epidemiological and financial data from the NTD programme of the Ghana Health Service. The key features of this costing model are its ease of use and the flexibility to extrapolate and predict periodic MDA costs for the elimination of LF. This LF-MDA cost modelling approach can be adopted and adapted by LF-endemic countries to support their national plans and fundraising.

This study has some key limitations. First, placing a value on the time spent by personnel on the MDA was difficult as detailed time records were not readily available. CHNs/CDDs time spent during the MDA programme was reported in total days spent in the field by the national NTD programme. Thus, they all spent 14 days in the field. Second, the estimation of unit cost per person treated was based on the national census figures for the endemic districts which in turn was used to estimate the MDA target populations for each district, due to indication from the LF SENTINEL study that there seems to be a much larger at-risk population compared to data from the national programme level [4]. In addition, another limitation is that we made assumptions about the number of people untreated and the success rates of E&T and T&T since there are possible outcomes like complete refusal to participate in the MDA and mop-up strategies by individuals. In the SENTINEL study, about 4% of participants (including some positives) refused all interventions and represented an interest group with the potential to sustain some level of transmission. Similarly, there is the likelihood that not all individuals who missed MDA were reached in this study, thus affecting the population estimates. This study does not provide a clear number of future MDA rounds saved by E&T and T&T intervention. However, this could be obtained through further statistical and mathematical modelling approaches to assess the number of MDAs saved through these strategies. Although the E&T and T&T methods may be limited to settings where MDA depends on the use of community registers, the registers provide opportunities for the optimal use of available data to further strengthen MDA programmes and is recommended where these are not already in place. Finally, the analyses were conducted based on information available at the time of the study. As such, it is possible that some districts may have progressed and stopped MDA over time. Despite these limitations, this study provides evidence on the cost of various proposed MDA approaches to guide programme options to eliminate LF in Ghana. The study even though conducted in Ghana, a lower-middle income country in Africa, presents a bouquet of cost scenarios for similar representative LF endemic countries in Africa.

## Conclusion

This study is one of the first comprehensive financial NTD programme costing studies of LF-MDA in Ghana in 20 years, the last financial costing having been done in 2002 [29]. We found that the costs of LF-MDA increase progressively annually. The findings set the financial basis for a comprehensive long-term investment decision for the elimination of LF in hotspot areas using the E&T and T&T strategies. The data presented is important for local and international policymakers for planning and forecasting health budgets towards addressing the end-game elimination challenges in hotspot districts. When faced with the prospect of requiring MDA for another 3–5 years, combining MDA with additional strategies will come with high costs but will pay off by improving treatment coverage, thus getting these districts to become LF-free in the least possible time.

## Supporting information

S1 Table LF-MDA cost categories for Ghana. (DOCX)

S2 Table LF-MDA Financial Costing (National, Regional and District Levels). (DOCX)

S3 Table Cost Assumptions. (DOCX)

S4 Table Projected LF-MDA eligible population by district from 2024–2026. (DOCX)

S5 Table Adjusted LF-MDA Treated Population coverage of 71% of eligible population for 2024–2026 by district. (DOC)

S6 Table Estimated LF-MDA Untreated Eligible Population of 29% for 2024–2026 by district. (DOC)

S7 Table Projected and Estimated LF-MDA financial cost per person (US$) for 2024–2026 by district. (DOC)

S8 Table Total financial cost of LF-MDA using adjusted population coverage rate of 71% (US$) by district. (DOC)

S9 Table Estimated financial cost of E&T mop-up strategy (US$) for 2024–2026 by district. (DOC)

S10 Table Estimated financial cost of NTD-programme-led T&T mop-up strategy (US$) for 2024–2026 by district. (DOC)

S11 Table Estimated financial cost of Health System Integrated T&T mop-up strategy (US$) for 2024–2026 by district. (DOC)

S12 Table Estimated financial cost of MDA approaches in US$ for 2024–2026. (DOCX)

## References

[1] WHO. Lymphatic filariasis [World Health Organisation website]. Who. int. World Health Organization: WHO; 2022. Available from: https://www.who.int/news-room/fact-sheets/detail/lymphatic-filariasis

[2] Biritwum NK, Frempong KK, Verver S, Odoom S, Alomatu B, Asiedu O, et al. Progress towards lymphatic filariasis elimination in Ghana from 2000–2016: Analysis of microfilaria prevalence data from 430 communities. Churcher TS, editor. PLOS Neglected Tropical Diseases. 2019 Aug 9;13(8):e0007115. doi: 10.1371/journal.pntd.0007115 31398203 PMC6709921

[3] Biritwum NK, Yikpotey P, Marfo BK, Odoom S, Mensah EO, Asiedu O, et al. Persistent “hotspots” of lymphatic filariasis microfilaraemia despite 14 years of mass drug administration in Ghana. Transactions of The Royal Society of Tropical Medicine and Hygiene. 2016 Dec;110(12):690–5. doi: 10.1093/trstmh/trx007 28938053

[4] de Souza DK, Otchere J, Sumboh JG, Asiedu O, Opare J, Asemanyi-Mensah K, Boakye DA, Gass KM, Long EF and Ahorlu CS (2022). Finding and eliminating the reservoirs: Engage and treat and test and treat strategies for lymphatic filariasis programs to overcome endgame challenges. Front. Trop. Dis. 3:953094. doi: 10.3389/fitd.2022.953094

[5] Amarillo MLE, Belizario VY Jr, Panelo CIA, Sison SAM, De Leon WU, Ramirez BL, et al. Cost of Mass Drug Administration for Filariasis Elimination in the province of Sorsogon, Philippines. Acta Medica Philippina. 2023 Jan 30;43(4).

[6] Goldman AS, Brady MA, Desir L, Direny A, Oscard R, Vely JF, et al. Costs of Integrated Mass Drug Administration for Neglected Tropical Diseases in Haiti. The American Journal of Tropical Medicine and Hygiene. 2011 Nov 1;85(5):826–33. doi: 10.4269/ajtmh.2011.10-0635 22049035 PMC3205627

[7] Kolaczinski JH, Robinson E, Finn TP. The Cost of Antibiotic Mass Drug Administration for Trachoma Control in a Remote Area of South Sudan. I. Ko A, editor. PLoS Neglected Tropical Diseases. 2011 Oct 11;5(10):e1362. doi: 10.1371/journal.pntd.0001362 22022632 PMC3191128

[8] Gedge LM, Bettis AA, Bradley MH, Hollingsworth TD, Turner HC. Economic evaluations of lymphatic filariasis interventions: a systematic review and research needs. Parasites & Vectors. 2018 Feb 1;11(1). doi: 10.1186/s13071-018-2616-z 29391042 PMC5793442

[9] Gyapong JO, Owusu IO, da-Costa Vroom FB, Mensah EO, Gyapong M. Elimination of lymphatic filariasis: current perspectives on mass drug administration. Res Rep Trop Med. 2018 Mar 6;9:25–33. doi: 10.2147/RRTM.S125204 ; PMCID: PMC6047620. 30050352 PMC6047620

[10] Mow M, Thean LJ, Parnaby M, Mani J, Rafai E, Sahukhan A, et al. Costs of mass drug administration for scabies in Fiji. Turner HC, editor. PLOS Neglected Tropical Diseases. 2022 Feb 3;16(2):e0010147 doi: 10.1371/journal.pntd.0010147 35113888 PMC8846527

[11] Gao B., Saralamba S., Lubell Y., White L. J., Dondorp A. M., & Aguas R. (2020). Determinants of MDA impact and designing MDAs towards malaria elimination. eLife, 9, e51773. doi: 10.7554/eLife.51773 32293559 PMC7185997

[12] Ncube MV, Chimbari MJ. A Cost Analysis of Options for Schistosomiasis Control Mda Programs Targeting Children Aged Five Years and Below in Umkhanyakude District of Kwazulu-natal Province, South Africa. Research Square; 2020. doi: 10.21203/rs.3.rs-64034/v1

[13] Yukich JO, Scott C, Silumbe K, Larson BA, Bennett A, Finn TP, et al. Cost-Effectiveness of Focal Mass Drug Administration and Mass Drug Administration with Dihydroartemisinin–Piperaquine for Malaria Prevention in Southern Province, Zambia: Results of a Community-Randomized Controlled Trial. The American Journal of Tropical Medicine and Hygiene. 2020 Aug 6;103(2_Suppl):46–53. doi: 10.4269/ajtmh.19-0661 32618249 PMC7416981

[14] Kyaw Shwe Sin, Delmas G Drake T, Celhay O., Wirichada Pan-Ngum, Sasithon Pukrittayakamee, et al. Estimating the programmatic cost of targeted mass drug administration for malaria in Myanmar. BMC Public Health. 2021 Apr 29;21(1). doi: 10.1186/s12889-021-10842-5 33926399 PMC8082869

[15] Turner HC. Health economic analyses of the Global Programme to Eliminate Lymphatic Filariasis. International Health. 2020 Dec 22;13(Supplement_1):S71–4. doi: 10.1093/inthealth/ihaa095 33349885 PMC7753169

[16] Drummond M. Methods for the economic evaluation of health care programmes. Oxford, United Kingdom; 4th Edition. New York, NY, USA: Oxford University Press; 2015.

[17] Ghana | ESPEN [ESPEN website]. Who. int. 2023. Available from: https://espen.afro.who.int/countries/ghana

[18] Levin HM, McEwan PJ, Belfield C, Bowden AB, Shand R. Economic Evaluation in Education: Cost-Effectiveness and Benefit-Cost Analysis. Sage Publications Inc; 2018.

[19] Toor J, Hamley JID, Fronterre C, Castaño MS, Chapman LAC, Coffeng LE, et al. Strengthening data collection for neglected tropical diseases: What data are needed for models to better inform tailored intervention programmes? Horstick O, editor. PLOS Neglected Tropical Diseases. 2021 May 13;15(5):e0009351. doi: 10.1371/journal.pntd.0009351 33983937 PMC8118349

[20] 2021 Mectizan Donation Program Annual Highlights [Internet]. Mectizan Donation Program. Available from: https://mectizan.org/news_resources/2021-mectizan-donation-program-annual-highlights

[21] Exchange Rate–Bank of Ghana [Internet]. Available from: https://www.bog.gov.gh/economic-data/exchange-rate/

[22] Ghana Statistical Service, Ghana 2021 Population and Housing Census General Report Vol 3B Age and Sex Profile. Service GS editor. Ghana Statistical Service. Ghana Statistical Service; 2021. Available from: https://statsghana.gov.gh/gsspublications.php?category=OTc2NDgyNTUzLjkzMDU=/webstats/p9r0796n5o#

[23] Division UNP. By Indicator | Pivot Table | Data Portal [Internet]. Population Division Data Portal. Available from: https://population.un.org/dataportal/data/indicators/59/locations/288/start/2019/end/2022/table/pivotbyindicator

[24] Ghana Statistical Service: Consumer Price Index Bulletin Ghana Statistical Services. statsghana.gov.gh. Available from: https://statsghana.gov.gh/nationalaccount_macros.php?Stats=MTE2MTIyMjQ5Ni41NjY=/webstats/7163p83s71

[25] OANDA Foreign Exchange Data Historical Currency Converter | OANDA. fxds-hcc.oanda.com. Available from: http://www.oanda.com/currency/historical-rates

[26] Ghana Statistical Service, +. Service, editor. Ghana Statistical Service. Ghana Statistical Service; 2021. Available from: https://statsghana.gov.gh/gssmain/fileUpload/pressrelease/2021%20PHC%20General%20Report%20Vol%203A_Population%20of%20Regions%20and%20Districts_181121.pdf

[27] Lee K. Systematic exchange rate variation: Where does the dollar factor come from? International Review of Economics & Finance. 2018 Jul;56:288–307.

[28] Turner HC, Toor J, Hollingsworth TD, Anderson RM. Economic Evaluations of Mass Drug Administration: The Importance of Economies of Scale and Scope. Clinical Infectious Diseases. 2017 Nov 8;66(8):1298–303. 10.1093/cid/cix1001 PMC5888956 29126255

[29] Goldman AS, Guisinger VH, Aikins M, Amarillo MLE, Belizario VY, Garshong B, et al. National Mass Drug Administration Costs for Lymphatic Filariasis Elimination. PLoS Neglected Tropical Diseases [Internet]. 2007 Oct 31 [cited 2020 Mar 25];1(1). Available from: https://www.ncbi.nlm.nih.gov/pmc/articles/PMC2041814/10.1371/journal.pntd.0000067 PMC2041814 17989784

[30] Baker MC, Krotki K, Sankara DP, Trofimovich L, Zoerhoff KL, Courtney L, et al. Measuring treatment coverage for neglected tropical disease control programs: analysis of a survey design. American Journal of Epidemiology [Internet]. 2013 Jul 15 [cited 2023 Sep 20];178(2):268–75. Available from: doi: 10.1093/aje/kws468 23860563

[31] de Souza DK, Yirenkyi E, Otchere J, Biritwum NK, Ameme DK, Sackey S, et al. Assessing Lymphatic Filariasis Data Quality in Endemic Communities in Ghana, Using the Neglected Tropical Diseases Data Quality Assessment Tool for Preventive Chemotherapy. Lammie PJeditor. PLOS Neglected Tropical Diseases. 2016 Mar 30;10(3):e0004590. doi: 10.1371/journal.pntd.0004590 27028010 PMC4814091

[32] Kastner RJ, Sicuri E, Stone CM, Matwale G, Onapa A, Tediosi F. How much will it cost to eradicate lymphatic filariasis? An analysis of the financial and economic costs of intensified efforts against lymphatic filariasis. PLoS Neglected Tropical Diseases [Internet]. 2017 Sep 26;11(9). Available from: https://www.ncbi.nlm.nih.gov/pmc/articles/PMC5630187/ doi: 10.1371/journal.pntd.0005934 28949987 PMC5630187

[33] Mulebeke R, Wanzira H, Bukenya F, Eganyu T, Collborn K, Elliot R, et al. Implementing population-based mass drug administration for malaria: experience from a high transmission setting in North Eastern Uganda. Malaria Journal. 2019 Aug 9;18(1). doi: 10.1186/s12936-019-2902-z 31399051 PMC6688214

[34] Downs PW, Bardin LE, McFarland DA. Modelling the dynamics of incentives in community drug distribution programs. Trends in Parasitology. 2014; 30(7): 317–319. 24793100 10.1016/j.pt.2014.04.001

[35] Otoo DD, Appiah-Agyekum NN, Adzei FA. Perceived determinants of implementation success of the neglected tropical diseases programme in Ghana: a qualitative study among programme officers. BMC Public Health. 2021; 21:2074. doi: 10.1186/s12889-021-12096-7 34763702 PMC8588665

