# Supplementary material for: Modelling the cost of engage & treat and test & treat strategies towards the elimination of lymphatic filariasis in Ghana
Source: PLoS Negl Trop Dis. 2024 May 24;18(5):e0012213. doi: 10.1371/journal.pntd.0012213 (PMC11156436; doi:10.1371/journal.pntd.0012213)
Supplement: S1 Table — (DOCX) [file pntd.0012213.s001.docx]

S1 Table: LF-MDA cost categories for Ghana

| Cost Category | Description |
| --- | --- |
| MDA Launch | This is the national launch of the LF-MDA programme activities. This includes the invitation of stakeholders in health including the media to announce the commencement of activities of the LF-MDA, targets hope to be achieved and educate the public on the importance of participation. |
| Social mobilisation | The involves the activities of gong-gong beaters, information centres (i.e., local radio stations) and community volunteers among others to spread information about the upcoming LF-MDA exercise at the regional and district levels. |
| Training | These are training activities done for the LF-MDA exercise, they include: Training of Trainers at the national level for regional disease control officers to further train district health officers; District Training Support by national level NTD officers to district level training; Regional Training Support to districts by regional disease control officers and programme officers to the districts; Training of CDDs by the district health managers and regional disease control officers; District Level Training by regional disease control officers and national NTD programme facilitators. |
| Personnel | This comprises of all personnel who conduct the LF-MDA activities: the national and regional NTD staff allowances and salaries, MDA allowances paid to CDDs and printing of CDDs name tags. |
| Supervision and Monitoring | This comprises of all related supervision and monitoring activities including national technical monitoring of drug distribution, regional monitoring of districts, district monitoring and sub-district monitoring by district health managers and NTD programme officers; and data management activities by national and regional NTD programme data officers including report writing of monitoring activities. |
| Drugs and Other Supplies distribution | This is mainly distribution cost was albendazole and ivermectin drugs distributed for use by CDDs and health workers in the implementation of the LF-MDA. Other supplies included protective plastic bags for registers, hand sanitizers, face masks (packs), ID tag holder for CDDs, Raincoat for CDDs. |
