# Supplementary material for: Modelling the cost of engage & treat and test & treat strategies towards the elimination of lymphatic filariasis in Ghana
Source: PLoS Negl Trop Dis. 2024 May 24;18(5):e0012213. doi: 10.1371/journal.pntd.0012213 (PMC11156436; doi:10.1371/journal.pntd.0012213)
Supplement: S2 Table — (DOCX) [file pntd.0012213.s002.docx]

S2 Table: LF-MDA Financial Costing (National, Regional and District Levels)

| Cost Category | Process |
| --- | --- |
| National Level | National level recurrent costs consist of the following: LF-MDA launch, training of trainers meeting, district training support, supervision & monitoring (technical), items for CDDs and health workers, salaries of 16 national NTD programme staff (i.e., salaries of national NTD Programme Staff were calculated based on number of days spent on the LF-MDA activities). |
| Regional Level | Regional level recurrent costs consist of the following: regional social mobilisation, regional training support to districts, regional monitoring support to districts and ten (10) regional NTD programme staff (i.e., share of salaries of regional NTD programme staff were calculated based on number of days spent on the LF-MDA activities). |
| District Level | District level specific recurrent costs consisted of the following: Training of 3,172 CDDs, district level training, social mobilization, LF-MDA implementation by CDDs, sub-district level monitoring and district level monitoring. |
