# Supplementary material for: Modelling the cost of engage & treat and test & treat strategies towards the elimination of lymphatic filariasis in Ghana
Source: PLoS Negl Trop Dis. 2024 May 24;18(5):e0012213. doi: 10.1371/journal.pntd.0012213 (PMC11156436; doi:10.1371/journal.pntd.0012213)
Supplement: S3 Table — (DOCX) [file pntd.0012213.s003.docx]

S3 Table: Cost Assumptions

| Category | Process |
| --- | --- |
| Population | The 2021 Population and Housing Census data was used to estimate the untreated population, excluding children under 5 years old |
|  | The average adult crude mortality rate for 2019-2022 (i.e., 7.4 per 1,000 population) according to UNDESA population division was used to adjust for population increases in subsequent years; |
|  | The estimate of 29% untreated population based on the LF SENTINEL study in Ahanta West was extrapolated to other districts |
| Financial Cost | 5-year average annual inflation rate i.e., 13.9% to estimate projected costs and costs per person to be reached in subsequent MDA rounds. |
|  | Estimates were provided for the 11 MDA districts in 2021, irrespective of their status over the following years. |
| Engage and Treat (E&T | 75.9% of the untreated population treated with the E&T mop-up strategy, as observed in the LF SENTINEL study in Ahanta West district; and |
|  | The same number of CDDs per the LF SENTINEL study (i.e., 120) was used for all districts with the assumption that this would not change over the rounds. |
| Test and Treat (T&T) | 24.1% of untreated population reached with the T&T mop-up strategy as observed in the LF SENTINEL study in Ahanta West district. |
|  | T&T is done through the NTD programme, using programme officers and all accompanying costs (per diem, transport, etc.). |
|  | T&T is integrated into the health-system with or without allowances paid to the CHNs |
