# Supplementary material for: Modelling the cost of engage & treat and test & treat strategies towards the elimination of lymphatic filariasis in Ghana
Source: PLoS Negl Trop Dis. 2024 May 24;18(5):e0012213. doi: 10.1371/journal.pntd.0012213 (PMC11156436; doi:10.1371/journal.pntd.0012213)
Supplement: S4 Table — (DOCX) [file pntd.0012213.s004.docx]

S4 Table: Projected LF-MDA eligible population by district from 2024-2026

| Regions | Districts | 2024 | 2025 | 2026 |
| --- | --- | --- | --- | --- |
| Bono | **Sunyani Municipal** | 217,862 | 229,316 | 241,372 |
|  | **Sunyani West** | 153,072 | 161,120 | 169,591 |
| Savannah | **Bole** | 136,580 | 147,312 | 158,887 |
|  | **Sawla-Tuna-Kalba** | 132,881 | 143,323 | 154,584 |
| Upper East | **Nabdam** | 59,108 | 62,806 | 66,736 |
| Upper West | **Lawra** | 67,164 | 71,568 | 76,261 |
|  | **Wa West** | 111,444 | 118,751 | 126,539 |
|  | **Wa East** | 105,122 | 112,015 | 119,361 |
| Western | **Ahanta West** | 169,891 | 177,974 | 186,441 |
|  | **Ellembelle** | 134,117 | 140,498 | 147,182 |
|  | **Nzema East** | 104,971 | 109,965 | 115,197 |
|  | **Total** | **1,392,211** | **1,474,647** | **1,562,149** |
