# Supplementary material for: Modelling the cost of engage & treat and test & treat strategies towards the elimination of lymphatic filariasis in Ghana
Source: PLoS Negl Trop Dis. 2024 May 24;18(5):e0012213. doi: 10.1371/journal.pntd.0012213 (PMC11156436; doi:10.1371/journal.pntd.0012213)
Supplement: S5 Table — (DOC) [file pntd.0012213.s005.DOC]

S5 Table: Adjusted LF-MDA Treated Population coverage of 71% of eligible population for 2024-2026 by district

| Regions | Districts | 2024 | 2025 | 2026 |
| --- | --- | --- | --- | --- |
| Bono | **Sunyani Municipal** | 154,682 | 162,814 | 171,374 |
|  | **Sunyani West** | 108,681 | 114,395 | 120,409 |
| Savannah | **Bole** | 96,972 | 104,591 | 112,810 |
|  | **Sawla-Tuna-Kalba** | 94,346 | 101,759 | 109,755 |
| Upper East | **Nabdam** | 41,966 | 44,592 | 47,383 |
| Upper West | **Lawra** | 47,686 | 50,813 | 54,145 |
|  | **Wa West** | 79,125 | 84,314 | 89,842 |
|  | **Wa East** | 74,636 | 79,531 | 84,746 |
| Western | **Ahanta West** | 120,623 | 126,362 | 132,373 |
|  | **Ellembelle** | 95,223 | 99,753 | 104,499 |
|  | **Nzema East** | 74,530 | 78,075 | 81,790 |
|  | **Total** | **988,470** | **1,047,000** | **1,109,126** |
