# Supplementary material for: Modelling the cost of engage & treat and test & treat strategies towards the elimination of lymphatic filariasis in Ghana
Source: PLoS Negl Trop Dis. 2024 May 24;18(5):e0012213. doi: 10.1371/journal.pntd.0012213 (PMC11156436; doi:10.1371/journal.pntd.0012213)
Supplement: S6 Table — (DOC) [file pntd.0012213.s006.DOC]

S6 Table: Estimated LF-MDA Untreated Eligible Population of 29% for 2024-2026 by district

| Regions | Districts | 2024 | 2025 | 2026 |
| --- | --- | --- | --- | --- |
| Bono | **Sunyani Municipal** | 63,180 | 66,502 | 69,998 |
|  | **Sunyani West** | 44,391 | 46,725 | 49,181 |
| Savannah | **Bole** | 39,608 | 42,720 | 46,077 |
|  | **Sawla-Tuna-Kalba** | 38,536 | 41,564 | 44,829 |
| Upper East | **Nabdam** | 17,141 | 18,214 | 19,354 |
| Upper West | **Lawra** | 19,477 | 20,755 | 22,116 |
|  | **Wa West** | 32,319 | 34,438 | 36,696 |
|  | **Wa East** | 30,485 | 32,484 | 34,615 |
| Western | **Ahanta West** | 49,268 | 51,612 | 54,068 |
|  | **Ellembelle** | 38,894 | 40,744 | 42,683 |
|  | **Nzema East** | 30,442 | 31,890 | 33,407 |
|  | **Total** | **403,741** | **427,648** | **453,023** |
