# Supplementary material for: Modelling the cost of engage & treat and test & treat strategies towards the elimination of lymphatic filariasis in Ghana
Source: PLoS Negl Trop Dis. 2024 May 24;18(5):e0012213. doi: 10.1371/journal.pntd.0012213 (PMC11156436; doi:10.1371/journal.pntd.0012213)
Supplement: S7 Table — (DOC) [file pntd.0012213.s007.DOC]

S7 Table: Projected and Estimated LF-MDA financial cost per person (US$) for 2024-2026 by district

| Regions | Districts | 2024 | 2025 | 2026 |
| --- | --- | --- | --- | --- |
| Bono | **Sunyani Municipal** | 0.63 | 0.72 | 0.82 |
|  | **Sunyani West** | 0.70 | 0.79 | 0.90 |
| Savannah | **Bole** | 0.92 | 1.04 | 1.19 |
|  | **Sawla-Tuna-Kalba** | 0.75 | 0.85 | 0.97 |
| Upper East | **Nabdam** | 1.47 | 1.68 | 1.91 |
| Upper West | **Lawra** | 1.10 | 1.26 | 1.43 |
|  | **Wa West** | 0.94 | 1.07 | 1.22 |
|  | **Wa East** | 1.06 | 1.20 | 1.37 |
| Western | **Ahanta West** | 0.59 | 0.67 | 0.77 |
|  | **Ellembelle** | 0.84 | 0.95 | 1.09 |
|  | **Nzema East** | 0.91 | 1.04 | 1.19 |
| National | **Average** | **0.83** | **0.95** | **1.08** |
