# Supplementary material for: Modelling the cost of engage & treat and test & treat strategies towards the elimination of lymphatic filariasis in Ghana
Source: PLoS Negl Trop Dis. 2024 May 24;18(5):e0012213. doi: 10.1371/journal.pntd.0012213 (PMC11156436; doi:10.1371/journal.pntd.0012213)
Supplement: S8 Table — (DOC) [file pntd.0012213.s008.DOC]

S8 Table: Total financial cost of LF-MDA using adjusted population coverage rate of 71% (US$) by district

| Regions | Districts | 2024 | 2025 | 2026 |
| --- | --- | --- | --- | --- |
| Bono | **Sunyani Municipal** | 98,100.75 | 117,714.57 | 141,249.89 |
|  | **Sunyani West** | 75,656.14 | 90,782.49 | 108,933.13 |
| Savannah | **Bole** | 88,881.45 | 109,286.45 | 134,375.94 |
|  | **Sawla-Tuna-Kalba** | 70,661.69 | 86,883.89 | 106,830.30 |
| Upper East | **Nabdam** | 61,704.86 | 74,745.29 | 90,541.62 |
| Upper West | **Lawra** | 52,584.02 | 63,876.72 | 77,594.60 |
|  | **Wa West** | 74,229.05 | 90,170.15 | 109,534.68 |
|  | **Wa East** | 78,750.03 | 95,662.04 | 116,205.99 |
| Western | **Ahanta West** | 71,155.74 | 84,976.71 | 101,482.20 |
|  | **Ellembelle** | 79,742.11 | 95,230.86 | 113,728.07 |
|  | **Nzema East** | 68,038.70 | 81,254.23 | 97,036.69 |
|  | **Total** | **819,504.54** | **990,583.39** | **1,197,513.11** |
