# Supplementary material for: Modelling the cost of engage & treat and test & treat strategies towards the elimination of lymphatic filariasis in Ghana
Source: PLoS Negl Trop Dis. 2024 May 24;18(5):e0012213. doi: 10.1371/journal.pntd.0012213 (PMC11156436; doi:10.1371/journal.pntd.0012213)
Supplement: S9 Table — (DOC) [file pntd.0012213.s009.DOC]

S9 Table: Estimated financial cost of E&T mop-up strategy (US$) for 2024-2026 by district

| Regions | Districts | 2024 | 2025 | 2026 |
| --- | --- | --- | --- | --- |
| Bono | **Sunyani Municipal** | 199,581.16 | 239,484.52 | 287,365.98 |
|  | **Sunyani West** | 140,227.94 | 168,264.49 | 201,906.53 |
| Savannah | **Bole** | 125,119.90 | 153,844.37 | 189,163.27 |
|  | **Sawla-Tuna-Kalba** | 121,731.51 | 149,678.08 | 184,040.50 |
| Upper East | **Nabdam** | 54,147.97 | 65,591.36 | 79,453.15 |
| Upper West | **Lawra** | 61,528.00 | 74,741.48 | 90,792.62 |
|  | **Wa West** | 102,092.49 | 124,017.41 | 150,650.83 |
|  | **Wa East** | 96,301.17 | 116,982.38 | 142,104.99 |
| Western | **Ahanta West** | 155,636.08 | 185,866.13 | 221,967.93 |
|  | **Ellembelle** | 122,863.47 | 146,727.92 | 175,227.69 |
|  | **Nzema East** | 96,163.26 | 114,841.58 | 137,147.89 |
|  | **Total** | **1,275,392.95** | **1,540,039.71** | **1,859,821.36** |
