# Supplementary material for: Modelling the cost of engage & treat and test & treat strategies towards the elimination of lymphatic filariasis in Ghana
Source: PLoS Negl Trop Dis. 2024 May 24;18(5):e0012213. doi: 10.1371/journal.pntd.0012213 (PMC11156436; doi:10.1371/journal.pntd.0012213)
Supplement: S10 Table — (DOC) [file pntd.0012213.s010.DOC]

S10 Table: Estimated financial cost of NTD-programme-led T&T mop-up strategy (US$) for 2024-2026 by district

| Regions | Districts | 2024 | 2025 | 2026 |
| --- | --- | --- | --- | --- |
| Bono | **Sunyani Municipal** | 298,558.63 | 408,406.25 | 490,061.15 |
|  | **Sunyani West** | 209,770.61 | 286,950.77 | 344,322.42 |
| Savannah | **Bole** | 187,170.10 | 262,359.34 | 322,590.62 |
|  | **Sawla-Tuna-Kalba** | 182,101.32 | 255,254.34 | 313,854.49 |
| Upper East | **Nabdam** | 81,001.36 | 111,856.59 | 135,495.86 |
| Upper West | **Lawra** | 92,041.33 | 127,460.79 | 154,833.69 |
|  | **Wa West** | 152,722.79 | 211,493.77 | 256,913.23 |
|  | **Wa East** | 144,059.41 | 199,496.54 | 242,339.52 |
| Western | **Ahanta West** | 232,820.04 | 316,967.83 | 378,534.23 |
|  | **Ellembelle** | 183,794.65 | 250,223.27 | 298,825.51 |
|  | **Nzema East** | 143,853.11 | 195,845.71 | 233,885.90 |
|  | **Total** | **1,907,893.35** | **2,626,315.21** | **3,171,656.63** |
