# Supplementary material for: Modelling the cost of engage & treat and test & treat strategies towards the elimination of lymphatic filariasis in Ghana
Source: PLoS Negl Trop Dis. 2024 May 24;18(5):e0012213. doi: 10.1371/journal.pntd.0012213 (PMC11156436; doi:10.1371/journal.pntd.0012213)
Supplement: S11 Table — (DOC) [file pntd.0012213.s011.DOC]

S11 Table: Estimated financial cost of Health System Integrated T&T mop-up strategy (US$) for 2024-2026 by district

| Regions | Districts | 2024 | | 2025 | | 2026 | |
| --- | --- | --- | --- | --- | --- | --- | --- |
|  |  | **H.S. integrated** | **H.S. integrated + allowances** | **H.S. integrated** | **H.S. integrated + allowances** | **H.S. integrated** | **H.S. integrated + allowances** |
| Bono | **Sunyani Municipal** | 180,230.70 | 181,588.19 | 216,265.21 | 217,894.11 | 259,504.31 | 261,458.88 |
|  | **Sunyani West** | 126,632.09 | 127,585.88 | 151,950.34 | 153,094.83 | 182,330.61 | 183,703.92 |
| Savannah | **Bole** | 112,988.86 | 113,839.89 | 138,928.33 | 139,974.74 | 170,822.88 | 172,109.50 |
|  | **Sawla-Tuna-Kalba** | 109,928.99 | 110,756.97 | 135,165.99 | 136,184.05 | 166,196.79 | 167,448.58 |
| Upper East | **Nabdam** | 48,898.04 | 49,266.34 | 59,231.93 | 59,678.06 | 71,749.74 | 72,290.15 |
| Upper West | **Lawra** | 55,562.53 | 55,981.03 | 67,494.89 | 68,003.26 | 81,989.79 | 82,607.33 |
|  | **Wa West** | 92,194.07 | 92,888.48 | 111,993.26 | 112,836.79 | 136,044.43 | 137,069.11 |
|  | **Wa East** | 86,964.26 | 87,619.27 | 105,640.31 | 106,435.99 | 128,327.15 | 129,293.70 |
| Western | **Ahanta West** | 140,546.33 | 141,604.91 | 167,845.41 | 169,109.62 | 200,446.95 | 201,956.71 |
|  | **Ellembelle** | 110,951.20 | 111,786.88 | 132,501.86 | 133,499.86 | 158,238.43 | 159,430.27 |
|  | **Nzema East** | 86,839.71 | 87,493.79 | 103,707.07 | 104,488.19 | 123,850.66 | 124,783.50 |
|  | **Total** | **1,151,736.78** | **1,160,411.61** | **1,390,724.62** | **1,401,199.48** | **1,679,501.73** | **1,692,151.65** |
