# Supplementary material for: Modelling the cost of engage & treat and test & treat strategies towards the elimination of lymphatic filariasis in Ghana
Source: PLoS Negl Trop Dis. 2024 May 24;18(5):e0012213. doi: 10.1371/journal.pntd.0012213 (PMC11156436; doi:10.1371/journal.pntd.0012213)
Supplement: S12 Table — (DOCX) [file pntd.0012213.s012.docx]

S12 Table: Estimated financial cost of MDA approaches in US$ for 2024-2026

| MDA Approach | 2024 | 2025 | 2026 |
| --- | --- | --- | --- |
| Current LF-MDA (71% of eligible population) | 819,504.54 | 990,583.39 | 1,197,513.11 |
| LF-MDA + E&T mop-up strategy alone | 2,094,897.49 | 2,530,623.09 | 3,057,334.47 |
| LF-MDA + E&T + T&T mop-up strategy integrated into health system | 3,246,634.27 | 3,921,347.71 | 4,736,836.21 |
| LF-MDA + E&T + T&T mop-up strategy integrated into health system (allowances paid) | 3,255,309.10 | 3,931,822.58 | 4,749,486.13 |
| LF-MDA + E&T + NTD-Programme-Led T&T mop-up strategy | 4,002,790.84 | 5,156,938.31 | 6,228,991.11 |
